# Porcine circovirus type 2 ORF3 protein induces apoptosis in melanoma cells

**DOI:** 10.1186/s12885-018-5090-2

**Published:** 2018-12-10

**Authors:** Marina Teras, Edda Viisileht, Merlis Pahtma-Hall, Airi Rump, Viiu Paalme, Pille Pata, Illar Pata, Christelle Langevin, Sirje Rüütel Boudinot

**Affiliations:** 1 Oncology, North Estonia Medical Centre, 19 J. Sütiste tee st, 13419 Tallinn, Estonia; 2 Department of Chemistry and Biotechnology, Tallinn University of Technology, 15 Akadeemia St, 12618 Tallinn, Estonia; 3 IVEX Lab OU, 15 Akadeemia St, 12618 Tallinn, Estonia; 4 Virologie et Immunologie Moléculaires, INRA, Université Paris-Saclay, 78350 Jouy-en-Josas, France

**Keywords:** Melanoma, Porcine circovirus type 2 (PCV2) ORF3, Apoptosis

## Abstract

**Background:**

The current treatment of malignant melanoma is limited by the lack of effective therapeutic approaches, and alternative treatments are needed. Proliferative diseases such as melanoma and other cancers may be treatable by virally-encoded apoptotic proteins that are targeted to rapidly multiplying cells. Caspase-dependent apoptosis, that is frequently used in chemotherapy, can boost the cell proliferation that caspase-independent cell death does not.

**Methods:**

In the current study, the porcine circovirus type 2 (PCV2), proapoptotic protein ORF3 was expressed in mouse and human cancer cell lines, and its apoptotic activity was assessed.

**Results:**

Quantitative assessment of the apoptotic cells by flow cytometry showed that apoptotic cell death was significantly increased in ORF3-expressing malignant cells, compared to ORF3 non-expressing cells. Our data show that PCV2 ORF3 induces apoptosis in a caspase-3 and -8 independent manner. ORF3 expression seems to cause an increase in abnormal mitosis in B16F10 melanoma cells by interacting with centrosomes and thereby disrupting the formation of the mitotic spindle. In addition, we show that ORF3 of PCV2 also exhibits significant anti-tumor effects in vivo. Although the expression of Regulator of G protein Signaling (RGS)-16 by recipient mice inhibited the development of grafted melanoma in vivo, it was not required for the antitumoral activity of ORF3.

**Conclusion:**

PCV2 ORF3 causes abnormal mitosis in rapidly dividing cells and increases the apoptosis of cancer cells. Apoptin might, therefore, be considered to develop future antitumoral strategies.

**Electronic supplementary material:**

The online version of this article (10.1186/s12885-018-5090-2) contains supplementary material, which is available to authorized users.

## Background

Apoptosis - also known as programmed cell death - maintains a healthy balance between cell survival and cell death in an organism. Apoptosis is triggered by the action of caspases, cysteine proteases that cleave key cellular structural components and activate degradation enzymes. Apoptosis actually has a critical impact effect on the malignant phenotype. Oncogenic mutations can lead to defects in apoptosis, and tumor initiation, progression or metastasis. Furthermore, mutations that disrupt apoptosis during cancer development may also decrease the sensitivity to cytotoxic antitumoral drugs. Cell death by apoptosis can also trigger immune responses, which can be useful in cancer treatment. The control of apoptosis, therefore, appears as an important approach to developing new cancer strategies.

The role of apoptosis in host-virus interaction is complex. During viral infection, apoptosis induces elimination of infected host cells to limit viral replication [1, 2]; in contrast, apoptosis of infected cells at the end of the viral life cycle may contribute to an efficient viral spread in the organism [3]. Interestingly, viral apoptins can mediate apoptosis selectively in oncogenically transformed cells with minimal toxicity for the normal cells of the host [4]. In tumor cells, such “apoptins” are phosphorylated and located mainly in the nucleus, whereas in normal cells they are not phosphorylated, reside in the cytoplasm, and can be readily neutralized [5]. Multiple studies have demonstrated the therapeutic potential of oncolytic viruses [6–9].

Several members of the Circoviridae family encode proteins with pro-apoptotic activity in tumor cells (*ie*, “apoptins”). The best-studied model for such oncolytic activity is probably the Chicken anemia virus (CAV) whose apoptin (VP3) can selectively induce apoptosis in H1299 non-small lung adenocarcinoma and chicken lymphoblast MDCC-MSB1 cells upon phosphorylation and translocation to the nucleus [10]. Porcine circovirus type 1 and 2 (PCV1 and 2) apoptin ORF3 also induces apoptosis in transformed cell [11]. An apoptotic activity has been reported for PCV2 ORF3 [2, 12], but its oncolytic activity remains unknown. It has been shown that PCV2 ORF3 induces apoptosis of infected porcine cells in a caspase-dependent manner, involving significant activation of both caspase-3 and caspase-8 [2]. However, in the porcine kidney (PK)-15 cell line PCV2 ORF3 protein induces apoptosis in cells by competitive binding to E3 ubiquitin-protein ligase Pirh2, thereby inhibiting its interaction with p53, leading to nuclear accumulation and activation of p53, and an increase of apoptosis [13].

In this study, we describe a pro-apoptotic activity of PCV2 ORF3 in human and mouse melanoma cell lines. ORF3 of PCV2 induced apoptosis in melanoma cells in vitro and in vivo. We also examined whether Regulator of G protein Signalling 16 (RGS16), which binds specifically to PCV2 ORF3 [14] and may be involved in oncogenic pathways [15, 16], modulates the effect of apoptin on melanoma growth. While the control of autologous tumor growth was impaired in RGS16 KO mice, our data suggest that the effect of ORF3 does not implicate RGS16.

## Materials and methods

### Mice

Two mouse strains were used in this study. Wild type (WT) *C57BL/6* J mice were initially obtained from Dr. Sulev Kuuse*,* Institute of Molecular and Cell Biology, University of Tartu*,* (Estonia). RGS16 knockout (KO) mice generated on C57BL/6 genetic background were obtained from Pr. Kirk Druey, NIAID, Bethesda (USA). All mice used in this study were grown in the fish facilities if Tallinn Technical university. Before experiments RGS16 KO mice were backcrossed 6 generation to our *C57Bl6*/J from Tartu University, to ensure an homogeneous background in WT and RGS16KO. The total number of the mice used in this study was 44 (20 for the comparison of tumor growth in WT and RGS16 KO mice, and 24 for the comparison of tumor growth in WT and RGS16−/− mice treated with PCV2ORF3 or control plasmid).

### Animal experiments

Animal handling and maintenance were performed according to the interdisciplinary principles and guidelines for the use of animals in research, testing and education (FELASA) prepared by the Ad Hoc Committee on Animal Research (The New York Academy of Sciences, New York, NY, USA). The animal experiments described in this study were authorized by the Ethical and Animal Welfare Committee of Estonia (University of Tartu, ERC PERMIT nr. 181 T-1). Mice were euthanized by cervical dislocation, according to our approved protocols.

### Cultivation of cell lines

B16F10 mouse melanoma cells and A375M human metastatic melanoma cells were cultured in DMEM (Dulbecco’s Modified Eagle Medium; PAA). THP-1 human acute monocytic leukemia cells were cultured in RPMI 1640 (Roswell Park Memorial Institute medium 1640; PAA). All media were supplemented with 12% fetal calf serum (FCS; Gibco) and 1% penicillin-streptomycin (Sigma). M2 human filamin-A-deficient melanoma cell line was cultured in MEM (Minimal Essential Medium; PAA), supplemented with 8% newborn calf serum (NBCS; Gibco), 2% FCS and 1% penicillin-streptomycin (Sigma). After transfection, cells were cultured in 1/1 mix of new medium and conditioned medium (culture supernatant collected from cell plates), greatly enhancing cell survival. The cells were maintained at 37 °C in a humidified incubator of 5% CO_2_ in 95% air and subcultured twice per week. B16F10, A375, and M2 cells were provided by Dr. Teet Velling (Department of Gene Technology, Tallinn University of Technology, Estonia), THP-1 cells were provided by Prof Jean-Marc Cavaillon (Institute Pasteur, Paris, France).

### Generation of the stable B16F10-luc cell line

B16F10 cell line expressing stably firefly luciferase (B16F10-luc) was generated using HIV1-based self-inactivating lentiviral vector system as described in [17]. Sequence encoding luciferase from the plasmid pGL3 (Promega) was cloned under the control of the strong constitutive human elongation factor 1-α promoter in a transfer vector. The virus was produced by transient transfection of the transfer vector with accessory plasmids in HEK293T cells and pseudotyped with VSV-G envelope protein. Viral supernatant was used to infect B16F10 cells from which single cell-derived positive clone was isolated.

### Isolation and cultivation of primary cells

RGS16 knockout (KO) and wild-type (WT) C57BL/6 mice were anesthetized using carbon dioxide and euthanized by cervical dislocation. Spleens from mice were removed and cell suspensions were obtained by pressing the spleens through a cell strainer in DMEM (PAA). Erythrocytes were lysed with ACK (Ammonium-Chloride-Potassium) lysis buffer (Lonza) for 10 min at room temperature. Then, cells were pelleted by centrifugation, washed twice with PBS and cultured in DMEM supplemented with 10% FCS (Gibco) at 37 °C in a humidified incubator of 5% CO_2_ in 95% air.

### Construction of expression plasmids

To generate the pcDNA-ORF3 expression construct, the ORF3 of PCV2 was PCR amplified from the purified PCV2 genome (GenBank, AF055392). The PCR products were cloned into mammalian expression vector pcDNA3.1 (Invitrogen) using the *Kpn*I*/Xho*I sites. Additionally, ORF3 was also cloned into the *Kpn*I/*Xho*I sites of pcDNA3.1-His plasmid (Invitrogen) and the coding region of mCherry, excised from the pBSK-mCherry E3 vector, was added using the *Xho*I/*Xba*I sites, thus giving rise the expression plasmid pcDNA3-his-ORF3-mCherry. To obtain the pCEP-GFP-RGS16 expression construct, the full-length cDNA of porcine RGS16 (GenBank accession EU271873) was RT-PCR-amplified from LPS-activated poPBMCs. The PCR products were cloned into the *Xho*I/*Eco*RI sites of the pCEP-GFP plasmid (BD Biosciences). All of the used PCR primers were designed on the basis of ORF3- and RGS16-encoding sequences and the appropriate plasmid sequences.

### Transfection of cells

The plasmid pcDNA-ORF3 was transiently transfected into B16F10, A375M and M2 melanoma cells using Lipofectamine ™ LTX (Invitrogen) in accordance with the manufacturer’s instructions. For comparison, pcDNA-ORF3 was also transiently transfected into THP-1 human acute monocytic leukemia cells. As a negative control, parallel transfections with empty pcDNA3.1 plasmid were carried out. The cells were seeded in six-well plates at a density of 5 × 10^5^ cells per well. For each well of cells, 2.5 μg of plasmid DNA was diluted in 500 μl Opti-MEM®I Reduced Serum Medium, 2.5 μl of PLUS™ Reagent was added, and the mixture was incubated at room temperature for 10 min. 10 μl of Lipofectamine™ LTX was then added into the DNA solution and DNA: Lipofectamine complex was allowed to form by incubating at room temperature for 30 min. 500 μl of the DNA-Lipofectamine™ LTX complex was added to each well and the cells were then maintained at 37 °C in a humidified incubator with 5% CO_2_ in 95% air. After 24–48 h of transfection, cells were harvested, transfection efficiency was determined (Additional file 1: Figure S1) and cells were used for various assays described below.

### Investigation of apoptosis by AnnexinV/PI staining flow cytometry assay

The extent of apoptosis was determined by flow cytometry using FITC-labelled annexin V and propidium iodide (PI). The cells that were transfected with either a pcDNA-ORF3 or an empty pcDNA3 plasmid were analyzed for apoptosis using annexin-V-FITC/PI double staining and subsequent flow cytometry at 24 and 48 h after transfection according to the manufacturer’s protocol. Approximately 1 × 10^6^ cells were double stained with annexin-V-FITC and propidium iodide using the annexin-V-FITC Apoptosis Detection Kit (Sigma). Transfected cells were washed with cold PBS, resuspended in 1× binding buffer and combined with annexin-V-FITC and propidium iodide. The cells were incubated at room temperature in the dark for 15 min. Fluorescence was detected using a fluorescence-activated cell sorter (FACS Calibur, BD Biosciences). A total of 30,000 cells were collected for each sample and analyzed using CellQuest software (Becton Dickinson). A homogenous population of malignant cells or primary cells was chosen according to their size and granularity; debris and duplicates of cells were eliminated by gating. All treatments were performed at least in triplicate and all experiments were carried out three times.

### Measurement of caspase activities

1. Caspase-3 and caspase-8 activities were measured first using a Caspase Colorimetric Assay Kit (Enzo) following the manufacturer’s instructions. In brief, ORF3-transfected B16F10 melanoma cells and WT mouse primary splenocytes were collected and lysed in chilled lysis buffer at 24 and 48 h post-transfection. The supernatant was removed, and the total protein concentration of each sample was quantified using Nanodrop™ spectrophotometer. Then, 150 μg of protein was diluted to 50 μl cell lysis buffer for each assay. 50 μl of 2× reaction buffer (containing 10 mM DTT) and 5 μl of the 4 mM IETD-*p*NA or DEVD-*p*NA (200 μM final concentration) substrate were added to each sample and incubated at 37 °C for 2 h. The optical densities were measured at 405 nm with an ELISA microtiter plate reader (Thermo Scientific Multiskan FC Microplate Photometer). The cells transfected with the empty pcDNA3 plasmid were used as negative control.
2. Caspase-3 activity was measured thereafter using intracellular staining in flow cytometry and an antibody that recognizes only cleaved and active form of Caspase-3. Since caspase-8 can be activated by autocleavage and can then trigger effector pro caspase-3 we determined the presence of a cleaved and active form of caspase-3 inside of B16F10 melanoma and PK15 cells.

### Cell death in the presence of caspase inhibitors

To reveal any underlying caspase-independent pathways induced by PCV2 ORF3 Q-VD-Oph, an irreversible pan-caspase inhibitor was added to the cells prior to treatment. Mouse melanoma B16F10 or porcine kidney 15 (PK15) cells were pretreated with 5 μM Q-VD-Oph (quinolyl-valyl-O-methylaspartyl-[− 2, 6-difluorophenoxy]-methyl ketone) (Sigma) for 30 min to inhibit caspases and then transfected with PCV2-ORF3 or control plasmid as described before. Transfection efficiency, cell viability, and morphology were investigated as previously described. After 48 h cells were harvested and apoptosis was investigated by AnnexinV/PI staining as described above (in Flow Cytometry assay section).

### Nuclear staining with 4′,6-diamidino-2-phenylindole

ORF3-transfected B16F10 cells grown on glass coverslips were washed 24 and 48 h post-transfection with PBS and subsequently fixed with 4% paraformaldehyde in PBS for 15 min. Fixed cells were then washed with PBS and incubated with 100 ng/ml DAPI (4′,6-diamidino-2-phenylindole; Sigma) in water for 30 min at room temperature in the dark. Stained slides were mounted using Fluoroshield Mounting Media (Sigma). The cells that were transfected with the empty plasmid were used as negative control. The nuclear morphology of the cells was examined by fluorescence microscopy. One hundred ORF3-positive and ORF3-negative control cells in mitotic phase were scored for normal/abnormal mitotic spindle formation.

### Indirect immunofluorescence assay

For the indirect immunofluorescence assay, previously transfected and paraformaldehyde-fixed B16F10 cells were washed with PBS containing 0.1% saponin (PBS-S). Non-specific immunoreactions were blocked with PBS-S containing 1% bovine serum albumin (BSA; PAA) at room temperature for 40 min. The cells were then washed three times with PBS-S. Primary antibody, mouse anti-α-tubulin monoclonal antibody IgG (Dako) were diluted 1:1000 in PBS-S and incubated with cells at room temperature for 2 h. After washing with PBS-S, the cells were incubated with FITC-conjugated anti-mouse IgG (Dako) diluted 1:1000 in PBS-S at room temperature and protected from light for 1 h. After three further washes, nuclei were stained with DAPI as described above. The cells were visualized by fluorescence microscopy.

### Co-expression of ORF3 and RGS16 in porcine peripheral blood mononuclear cells

Porcine peripheral blood mononuclear cells (poPBMC) were isolated from conventionally reared Yorkshire pig blood by density-gradient centrifugation on Ficoll-Paque (Amersham Pharmacia Biotech) and cultivated directly in coverslip that was placed in the bottom of 24 well plates. Porcine PBMCs were cultured in RPMI 1640 medium (BioWhittaker) supplemented with 20 mM HEPES, 2 mM L-glutamine, 200 IU penicillin ml^− 1^, 100 μg streptomycin ml^− 1^, 0.5 μM 2-Mercaptoethanol and 5% FCS at 37 °C in a humidified incubator with 5% CO_2_ in 95% air. The cells were activated with LPS (lipopolysaccharide) from *Escherichia coli* serotype 0111: B6 (2,5 μg ml^− 1^; Sigma).

LPS-activated poPBMCs were then transiently transfected with pcDNA3.1-His-ORF3-mCherry in combination with pCEP-GFP-RGS16. The cells were seeded on glass coverslips placed in the bottom of six-well plates and transfected using FuGene® 6 reagent (Roche), following the manufacturer’s instructions. The cells were harvested 48 h after transfection.

The endogenous expression of RGS16 and ORF3 in poPBMCs was determined by indirect immunofluorescence assay using a rabbit-human RGS16-specific polyclonal antibody (Aviva Systems Biology) and a mouse monoclonal antibody recognizing the 6× His (Clontech) tag of the histidine-tagged ORF3 construct, respectively. Porcine PBMCs were fixed in 4% paraformaldehyde and non-specific immunoreactions were blocked by using PBS-S containing 1% BSA. After incubation with the primary antibody, the cells were stained with FITC-labeled antibody to rabbit Ig (Dako) or with monoclonal anti-mouse Igκ coupled to Texas red (Serotech). The cells’ nuclei were stained with the fluorescent dye Hoechst 33258 (Sigma). The cells were then visualized by fluorescence microscopy.

### Fluorescence microscopy

All fluorescence microscopy was performed using an Axioplan II Imaging fluorescence microscope equipped with appropriate filter sets, an Axiocam charge-coupled device camera and Axiovision software (Carl Zeiss Light Microscopy). Digital images were processed using Adobe Photoshop version CC software.

### Subcutaneous grafts of B16F10-luc melanoma cells in RGS16 knockout mouse model

Exponentially growing B16F10-luc cells expressing stably firefly luciferase were harvested and injected subcutaneously into the right flank of 6–7-week-old female WT and RGS16 KO mice (1 × 10^6^ cells per implant). Tumor development was monitored by luciferase activity using IVIS Lumina II imaging system (Caliper Life Sciences, now PerkinElmer). Before the scan, mice were shaved, injected intraperitoneally with D-luciferin (Regis Technologies, Illinois, USA) at 150 mg/kg body weight and dozed with 2.5% isoflurane. Sequential images were taken at 2 min intervals to determine the peak value of the luciferase signal. After 18 days mice were sacrificed, and the tumors were isolated and weighed.

### In vivo induction and treatment of melanoma

For in vivo induction of melanoma, 8–10-week-old WT and RGS16 KO C57BL/6 mice were injected subcutaneously into the derma of the right side of the back with 5 × 10^4^ B16F10 cells in 50 μl PBS. After tumor transplantation, its growth was followed at least every second day. After 18 days or with the appearance of either a dark pigmentation or a solid tumor on the melanoma inoculation site, 10 μg of pcDNA3-ORF3 expression plasmid (dissolved in 10 μl PBS) was injected into the tumor site. The control mice were injected with the same volume of the empty pcDNA3.1 plasmid. The length of the tumor was measured along the imaginary longitude of the back and its width was measured in the direction of the latitude with an accuracy of 0.5 mm using a digital caliper. Subcutaneous tumor volume was calculated using the following formula: V = π/6·ƒ·(length·width)^3/2^, where ƒ = 1.69 (+/− 0.3) for male mice and f = 1.58 (+/− 0.1) for female mice [18]. Mice were sacrificed at day 28 after B16F10 melanoma cells injection, and all primary tumors were excised and weighed.

### Statistical analysis

The statistical significance was determined using Student’s t-test. The significance level was set at a *p*-value of 0.05.

## Results

### PCV2 ORF3 induces apoptosis in tumor cell lines

In order to assess the pro-apoptotic activity of PCV2 ORF3 in cancer cells, several tumor cell lines were transiently transfected with an expression plasmid encoding ORF3 and were observed during the following 3 days. We selected B16F10 mouse melanoma cells, metastatic A375M human melanoma cells, filamin-A-deficient M2 human melanoma cells, and THP-1 human acute monocytic leukemia cells.

**Fig. 1:**
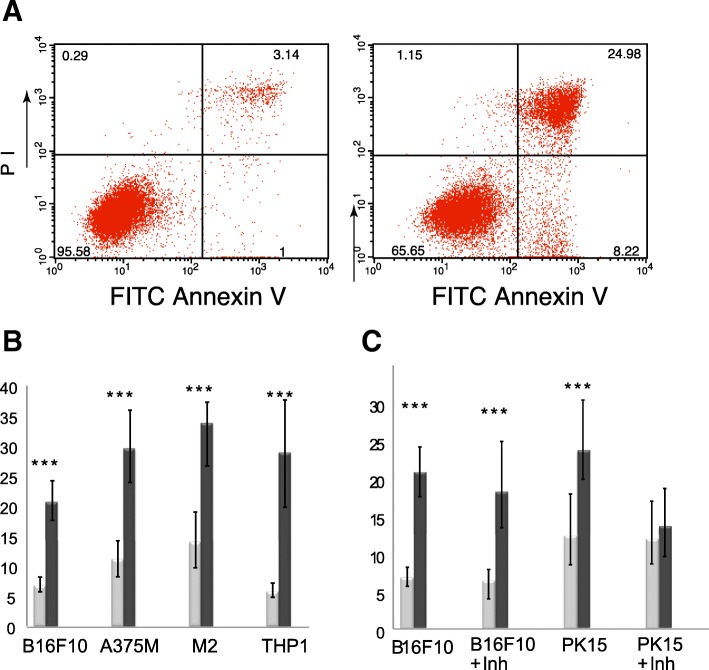
PCV2 ORF3 overexpression induces apoptosis in cancer cells. **a** Representative FACS analysis of apoptosis in human monocytic cell line THP-1, transfected with either empty pcDNA3.1 (left), or pcDNA3-ORF3 (right). Cells were stained by annexin-V-FITC (x-axis) and propidium iodide (y-axis) and analyzed at 48 h post-transfection by flow cytometry. Annexin-V-FITC single-positive cells (annexin+/PI-) correspond to early apoptotic cells, double-positive cells (annexin+/PI+) to late apoptotic, while double-negative cells (annexin-/PI-) represent non-apoptotic cells, and PI single-positive cells (annexin-/PI+) necrotic cells. In each quadrant, the percentage of cells out the FSC/SSC gate set-up for each cell line, is indicated. **b** Proportion of apoptotic cells (annexin+/PI- and annexin+/PI+) among cells overexpressing ORF3 (dark grey) or in controls (light grey); B16F10 mouse melanoma cells, metastatic A375M human melanoma cells, filamin-A-deficient M2 human melanoma cells, and THP-1 human acute monocytic leukemia cells were transfected and analyzed **c**. Caspase inhibition assay on B16F10 mouse melanoma cells and Porcine kidney 15 (PK15) cells. Cells were treated with pan-caspase inhibitor, then transfected with a plasmid expressing ORF3 or with pcDNA3-ORF3. Percentages of apoptotic cells (annexin+/PI- and annexin+/PI+) were quantified in cells overexpressing ORF3 (dark grey) and in controls (light grey). Error bars indicate standard deviations from 6 analyses. ***: Student test *p* < 0.005

The fraction of apoptotic cells was measured by flow cytometry 48 h after transfection using annexin-V-FITC and propidium iodide double staining. Annexin-V single-positive cells (annexin+/PI-) were considered as early apoptotic, double-positive cells (annexin+/PI+) as late apoptotic, while double-negative cells (annexin-/PI-) and PI single-positive cells (annexin-/PI+) representing non-apoptotic and necrotic cells, respectively. Cells transfected with empty pcDNA3 were used as a negative control. The rate of apoptosis in three melanoma cell lines and monocytic leukemia cell line all overexpressing ORF3 was significantly higher than in ORF3-negative cells, transfected with empty plasmid (Fig. 1).

These results show that PCV2 ORF3 induces apoptosis in cancer cells, and therefore appears to function similarly to its homologs in other circoviruses.

B16F10 mouse melanoma cells were selected for further studies on the basis of their higher transfection efficiency and sensibility to ORF3-induced apoptosis.

### PCV2 ORF3 expression elicits nuclear morphological changes characteristic of apoptosis in B16F10 cells

We also examined nuclear morphology in B16F10 mouse melanoma cells that were transfected with the ORF3 expression plasmid or with an empty pcDNA3 vector as a control. Nuclear morphology was analyzed by fluorescence microscopy of cells stained by the fluorescent DNA-binding agent DAPI at 24 and 48 h post-transfection.

**Fig. 2:**
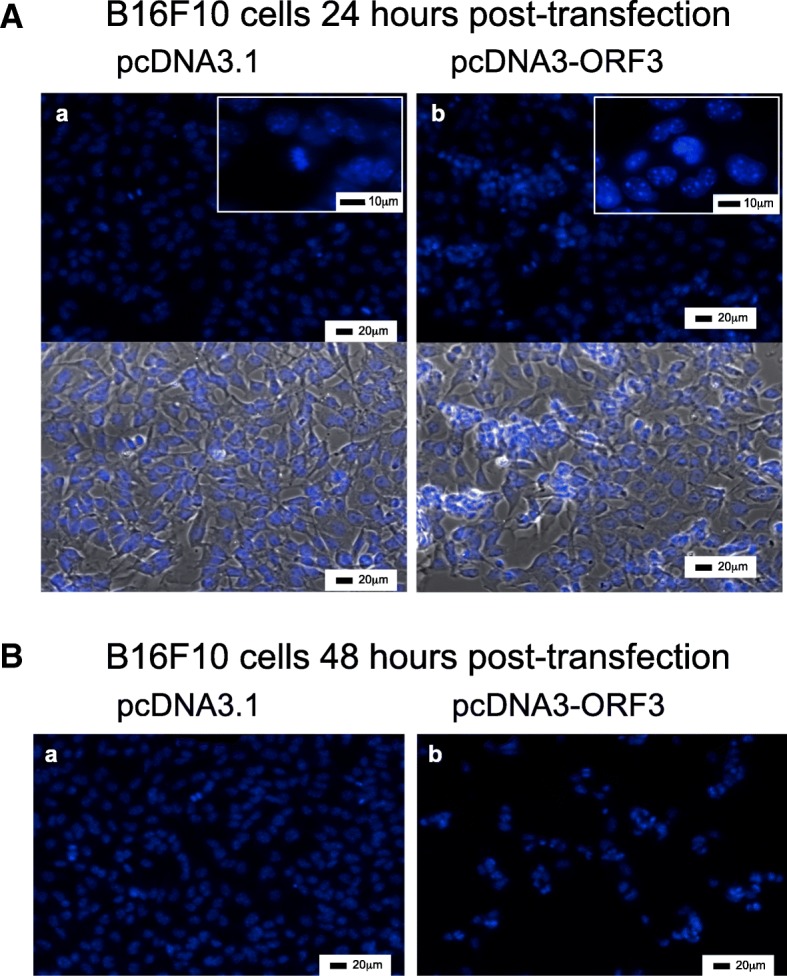
PCV2 ORF3 overexpression causes Abnormal Mitosis. B16F10 cells 24 h (A) or 48 h (B) post-transfection. B16F10 cells were transfected with either a pcDNA3.1 empty plasmid (**a**) or pcDNA-ORF3 plasmid (**b**), fixed and stained with DAPI (blue). Lower panels in Aa and Ab show pictures with phase contrast. Representative pictures of two independent experiments. Scale bars (20 or 10μm) are represented in each panel

No obvious nuclear morphological changes were observed in ORF3-negative control cells (Fig. 2Aa), showing low fluorescence intensity and homogeneous nuclear chromatin. In contrast, ORF3-transfected B16F10 cells displayed intense blue fluorescence and typical apoptotic morphological features, including nuclear condensation and nuclear fragmentation (Fig. 2Ab). In addition, cell proliferation of ORF3-transfected cells was reduced compared with control cells at 48 h post-transfection (Fig. 2B).

DAPI staining demonstrated that ORF3 expression elicited nuclear morphological changes characteristic of apoptosis in B16F10 cells, confirming that PCV2 ORF3 indeed induces cell death through apoptosis.

### ORF3 of PCV2 causes abnormal mitosis in B16F10 cells

The impact of PCV2 ORF3 overexpression on mitosis of B16F10 mouse melanoma cells was also studied. Endogenous tubulin was stained 48 h post-transfection with a FITC-labeled anti-α-tubulin antibody and DNA was stained with DAPI. One hundred ORF3-positive and ORF3-negative control cells in mitotic phase were scored for normal/abnormal mitotic spindle formation by fluorescence microscopy.

**Fig. 3:**
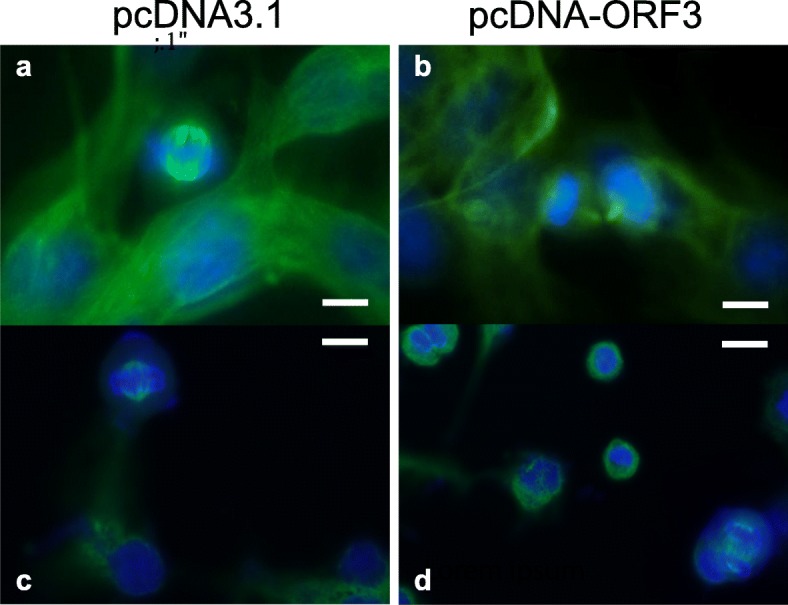
PCV2 ORF3 overexpression induces nucleus fragmentation. B16F10 cells were transfected with either pcDNA3.1 empty plasmid (**a** and **c**) or pcDNA-ORF3 plasmid (**b** and **d**) and fixed 48 h post-transfection. Endogenous tubulin was stained with a FITC-labeled anti-α-tubulin antibody (green) in panels **a** and **b**. DNA was stained with DAPI (blue). Scale bars 5μm

Most ORF3-negative B16F10 cells in the mitotic phase displayed normal, i.e. symmetric and bipolar mitotic spindle formation with only 6% of abnormality (Fig. 3, Additional file 2: Movie). B16F10 cells expressing ORF3 seems to display chromosome misalignment, disrupted mitotic spindles and abnormal mitosis at increased frequently (18%) These data would suggest that ORF3 expression causes an increase in abnormal mitosis formation in B16F10 cells that was not observed in ORF3-negative control cells.

### PCV2 ORF3 induces apoptosis in B16F10 cells through a Caspase-8 and Caspase-3 independent pathway

In order to characterize the apoptotic cell signaling pathway induced by PCV2 ORF3, activation of initiator caspase-8 and effector caspase-3 were examined in ORF3-transfected B16F10 mouse melanoma cells and WT mice primary splenocytes. Cells transfected with the empty pcDNA3.1 were used as negative control. Cell lysates were analyzed 24 and 48 h post-transfection for IETDase and DEVDase activity using specific colorimetric substrate IETD-*p*NA (caspase-8) or DEVD-*p*NA (caspase-3). B16F10 cells expressing ORF3 did not show any significant increase of caspase-8 or caspase-3 activity, compared with those transfected with an empty plasmid (Additional file 3: Figure S2).

**Fig. 4:**
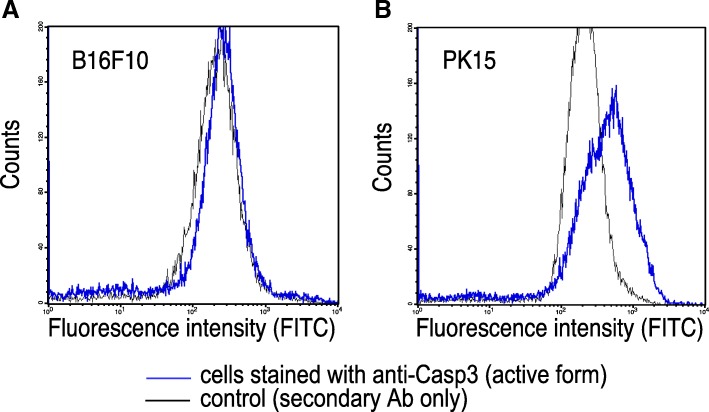
Lack of expression of the active form of caspase 3 in B16F10 cells transfected with ORF3. B16F10 (**a**) and PK15 (**b**) cells overexpressing ORF3 were stained intracellularly with an anti-caspase3 (active form) mAb (blue line). Cells stained with the secondary (anti-mouse Igκ) Ab are shown as negative controls (black line)

To reveal any underlying caspase-independent pathways induced by PCV2 ORF3 Q-VD-Oph, an irreversible pan-caspase inhibitor was added to mouse melanoma (B16F10) and porcine kidney 15 (PK15) cells 30 min prior transfection with PCV2-ORF3 encoding or empty plasmid. The presence of caspase-3 activities was measured thereafter using intracellular staining in flow cytometry and an antibody that recognizes only cleaved and active form of caspase-3. Since caspase-8 can be activated by autocleavage and it can then trigger effector procaspase-3, we determined the presence of a cleaved and active form of caspase-3 inside B16F10 and PK15 cells. Using intracellular staining and flow cytometry we could detect caspase 3 activity in PK15 cells and not in B16F10 melanoma cells (Fig. 4).

These results indicate that PCV2 ORF3 induces apoptosis in B16F10 and in mouse primary splenocytes in a caspase-8- and − 3-independent manner (Additional file 3: Figure S2) while in porcine kidney cell line PCV2 ORF3 induces apoptosis at least in caspase-3 dependent way.

### PCV2 ORF3 exhibits anti-tumor effects in vivo

Melanoma growth was assessed in 7–8-week-old mice after subcutaneous injection of B16F10-luc melanoma cells expressing stably and constitutively firefly luciferase. A clonal B16F10-luc cell line had been generated by lentiviral transduction and subsequent subcloning [17]. We took advantage of the histocompatibility of this cell line and regulator of G protein 16 (RGS16) knock out mice to test the impact of RGS16 on the tumor growth.

Tumor progression was monitored for 28 days (tumor transplantation set-up as day 0), then mice were sacrificed, and the tumors weighed. The effect of ORF3 on tumor development was tested by injection of ORF3 expression plasmid or a control empty pcDNA3.1 plasmid directly into the tumor region as soon as an area of dark pigmentation or a solid nodular tumor appeared at the site of injection. Pigmentation became first visible at day 15 after tumor implantation and by day 18 it was possible to measure the tumor by caliper. In literature, several methods have been proposed for the calculation of tumor size, most often based on the measurement of the three dimensions of the tumor. We followed the growth of tumors by measuring two dimensions - the height and width of the tumor using an empirical formula developed by Feldman [18]. The final tumor weight is in good correlation with tumor volume calculated according to this empirical formula (*R* = 0.88).

**Fig. 5:**
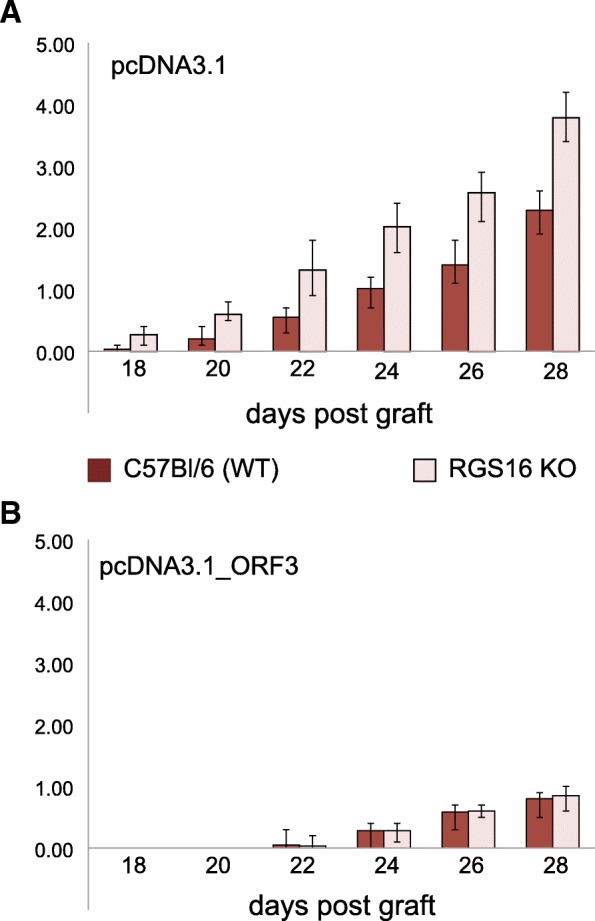
Tumor growth is inhibited by ORF3 in WT and RGS16 KO mice. **a** Growth dynamics of B16F10 tumor wild-type (WT) mice (dark columns, *n* = 6) or in RGS16 KO (light columns, *n* = 6), treated with pcDNA3.1. **b** Growth dynamics of B16F10 tumor in wild-type mice (dark, *n* = 6) or RGS16 KO mice (light, *n* = 6), treated with pcDNA-ORF3. The length and width of the tumor were measured in the direction of the latitude with an accuracy of 0.5 mm using a digital caliper and calculated as described in material and methods section. In the Y axes are shown melanoma volume V (cm^3^), calculated according to Feldman [18]. Error bars indicate standard deviation

A significant difference in tumor growth between pcDNA-ORF3 and pcDNA3.1 treated groups was observed in the following weeks (compare Fig. 5a and b): the visible appearance of tumors occurred in average at day 18 and 25 in control and ORF3 treated mice, respectively. Additionally, the end weight of tumors was much lower when the ORF3 expression plasmid was injected (Fig. 5a and b).

Hence, overexpression of ORF3 in the tumor reduced the growth of the melanoma in vivo.

### The expression of RGS16 by the tissues surrounding the tumor controls its growth but is not required for the anti-tumoral effect of ORF3 in vivo

We have previously shown that PCV2 ORF3 interacts with the Regulator of G protein Signalling (RGS)-16 [14]. RGS16 is involved in the regulation of signaling via several GPCRs, including chemokine receptors, and plays an important role in the activation of the antiviral response [19]. RGS16 also contributed to the translocation of ORF3 to the cell nucleus in activated cells co-transfected with RGS16 and ORF3 [14]. We, therefore, tested the idea that RGS16 might be involved in the induction of apoptosis by PCV2 ORF3. For this purpose, we used the in vivo melanoma model described above.

B16F10-luc melanoma cells expressing stably luciferase were injected subcutaneously into WT and RGS16 KO mice, and tumor progression was monitored for 28 days. Mice were then sacrificed and tumors weighed.

**Fig. 6:**
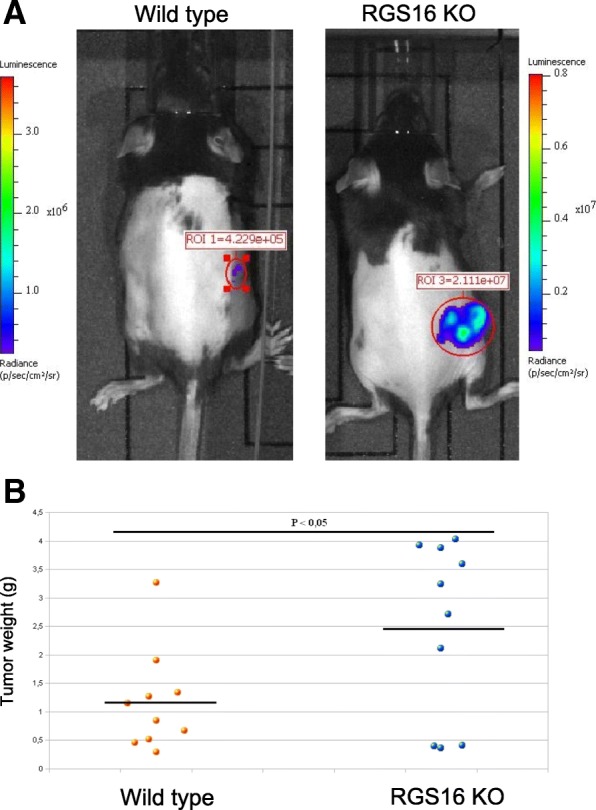
Tumor growth in enhanced in RGS KO mice. **a** Bioluminescence imaging of B16F10-luc subcutaneous grafts in WT and RGS16 KO mice at 8 days post-injection. Total flux (radiance) of the ROI (region of interest) of WT = 4229 · 10^5^ (p/s, photons per second). Total flux of the ROI of RGS16 KO = 2111 · 10^7^ p/s. Exposure time 3 min. **b** Melanoma graft tumor weight (g), isolated from female C57BL6 WT mice (*n* = 10) and RGS16 KO mice (*n* = 10) 21 days after cell engraftment. The difference in tumor weight between WT and RGS16 KO mice was statistically significant (*p* < 0.05) regardless of vast individual variability

Strikingly, RGS16 expressed by the recipient mouse controlled tumor development since larger primary tumors developed in RGS16 KO mice compared to WT (Fig. 6a). The difference in tumor weight between WT and RGS16 KO mice was statistically significant (*p* < 0.05) in spite of high individual variability (Fig. 6b). Thus, RGS16 is involved in the immune response against tumors, possibly by affecting immune cell activation and infiltration into the site of the tumor or influencing the local milieu in the tissue. However, the tumor development started at the same time both in WT and RGS16 KO mice.

Importantly, we observed that the decrease in tumor growth induced by the overexpression of ORF3 was still observed in RGS16 KO mice, demonstrating that an RGS16 expression around the tumor is not required for ORF3 antitumoral activity. It also suggests that the mechanisms through which ORF3 control the tumors are predominant over the impact of the RGS16 ^+^/_+_ cellular environment of the tumor (Fig. 6a and b).

Altogether, our data show that ORF3 mediates an antitumoral effect in vivo, most likely by inducing apoptosis of cancer cells through mechanisms independent of the expression of RGS16 in the host tissues.

## Discussion

We report here a pro-apoptotic activity of the PCV2 ORF3, a homolog of the apoptin of the circovirus chicken anemia, which has been previously reported to induce cell death in human cancer cells [20, 21]. Our results are consistent with another study describing a pro-apoptotic capacity of PCV2 ORF3 in porcine kidney cells [2, 22].

The rate of apoptosis in different melanoma cell lines overexpressing ORF3 was significantly higher than in ORF3-negative control cells, indicating that PCV2 ORF3 is able to induce apoptosis in malignant melanoma cells at least in vitro. THP-1 cells, which divide faster than melanoma cells, when overexpressing ORF3, also show a higher rate of apoptosis, suggesting that PCV2 ORF3-induced apoptosis might be generally facilitated in rapidly dividing cancer cells. Similar results have been observed earlier with CAV apoptin, which can induce programmed cell death in human transformed and malignant cells but fails to induce cell death in normal cells [23]. In normal cells, CAV apoptin is predominantly localized to the cytoplasm where it is efficiently degraded by the proteasome, whereas in transformed cells it localizes to the nucleus [24, 25]. Similarly, PCV2 ORF3 contains a putative nuclear localization signal (NLS) and a nuclear export signal (NES) and shows expected localization pattern.

Caspases play a critical role in the induction of apoptosis and are responsible for the morphological changes characteristic of apoptosis [26]. Previous studies have demonstrated that PCV2 ORF3 induces apoptosis in PK-15 cells and poPBMCs through the extrinsic pathway via sequential activation of caspase-8 and-3 [2]. In the current work, no significant differences in caspase-8 and-3 activity could be detected between ORF3-expressing and non-expressing B16F10 cells using a well-established assay, which suggests that apoptosis might not be induced here via the same pathway. These results indicate that PCV2 ORF3 induces apoptosis in cancer cells most probably in a caspase-independent manner. Caspase-independent cell death has been described in cervical cancer, where apoptosis was induced by oxidative stress-mediated activation of p53 and p38 with selenium [27].

Q-VD-OPh is a potent, cell-permeable inhibitor of caspase activity that has increased selectivity for the caspases over other cysteine proteases. The addition of Q-VD-OPh prevents the maturation of caspase-3 subunit p19 into the fully active p17, which explains the lack of PARP1 cleavage and inhibition of apoptosis under these conditions.

In fact, several studies suggest that PCV2 ORF3 protein-induced apoptosis may occur through a caspase-independent, p53-dependent pathway. The ORF3 protein has been shown to induce apoptosis in PK-15 cells by competitive binding to the E3 ubiquitin-protein ligase Pirh2, over at the expense of p53 binding. The ORF3-mediated inhibition of the p53-Pirh2 interaction has been proposed to lead to nuclear accumulation and activation of p53 and may increase the propensity of the cells to undergo apoptosis [13]. Apoptotic dysregulation is a hallmark of melanoma pathogenesis and chemoresistance. Mutations in p53 are seldom observed in melanoma and they are not critical for tumor development. Moreover, the presence of intact p53 can even increase the rate of apoptotic and autophagic cells [28]. Since p53 is often structurally preserved, but functionally defective due to loss of CDKN2A in melanoma, restoration of p53 function in melanoma could be a potential therapeutic strategy [29]. Therefore, the ORF3-mediated inhibition of the p53-Pirh2 interaction might represent an attractive mechanism for activating wild-type p53 in tumors by an anticancer agent. Since approximately half of all human tumors carry a mutation in the p53 gene, it is important to note that p53-dependent treatment strategies are useless against those cancer types.

Viruses can also encode caspase inhibitors as for example p35 (a baculovirus gene product) that may prevent cell death induced by various stimuli. Expression of p35 during viral infection may result in the inhibition of several caspases and make cells resistance to cell death [30]. PCV2 genome also encodes a protein with an anti-apoptotic activity called ORF4 that is smaller and overlaps with ORF3 in the same direction. Previous results have indicated that the ORF4 protein may play an important role by restricting ORF3 transcription thereby preventing virus-induced apoptosis [31]. It may be possible that in some host cells - PCV2 virus is able to infect only limited species and cell lines included PK15 - both proteins ORF3 and ORF4 are expressed to regulate cell death in the host.

Induction of apoptosis by PCV2 ORF3 in B16F10 cells was further confirmed by typical apoptotic morphological features, such as nuclear shrinkage and nuclear fragmentation. We also observed that cells expressing ORF3 show abnormal division figures. Interestingly, the localization of ORF3 in porcine PBMC transfected with an overexpression plasmid for an ORF3-mCherry fusion protein suggests that it might be coupled to centrosomes (Additional file 4: Figure S3), which could explain its impact on mitosis. Centrosomes have a crucial role in the formation of bipolar mitotic spindles, which are essential for accurate chromosome segregation into two daughter cells during cytokinesis. Cells with aberrant mitotic spindle formation fail to undergo cytokinesis, that, in turn, triggers the checkpoint response involving the p53, eventually leading to cell death [32]. Therefore, results presented here suggest that PCV2 ORF3 may cause apoptosis in cells through induction of abnormal mitoses by interacting with centrosomes and thereby disrupting mitotic spindle formation. However, further studies are required to elucidate the exact molecular mechanisms of ORF3-induced apoptosis.

We also showed that ORF3 can inhibit tumor growth in vivo, in a mouse model of melanoma graft. In mice injected with pcDNA-ORF3, the development of melanoma was delayed, the tumor growth was lower, and the final weight was significantly smaller compared to mice injected with the empty pcDNA3.1 vector.

Since ORF3 of PCV2 interacts directly with the RGS16 [14] and a number of studies have shown a role for RGS16 in the pathogenesis of several cancers [33–35], we tested whether RGS16 might be involved in the ORF3-mediated inhibition of melanoma growth. RGS16 KO mice developed larger primary tumors than WT mice after injection of melanoma cells, indicating that RGS16 expressed in the tissues surrounding the tumor has a protective effect [36]. However, our experiments show clearly that this expression of RGS16 is not necessary for the antitumoral activity of ORF3, indicating that the mechanisms are distinct.

## Conclusion

Taken together, our results show that PCV2 ORF3 causes non-bipolar mitosis in rapidly dividing cells and induces apoptosis. This antitumoral activity was observed in vitro and in vivo and is apparently independent of caspase 3 and caspase 8 and uses distinct mechanisms from those triggered by the host RGS16. We hope that the development of antitumor therapies might benefit from the understanding of the apoptin-dependent antitumoral activity.

## Additional files

Additional file 1: **Figure S1.** Transfection efficiency. To find out the most effective way for overexpression of recombinant proteins DNA was introduced into all cell lines by transfection with Lipofectamine (LTX) or polyethyleneimine (PEI). Consequently, the DNA: LTX and DNA: PEI complex penetrate cell membranes and recombinant proteins are produced. After 24–48 h of transfection, cells were harvested, and transfection efficiency of every experiment was determined using parallel wells that were transfected only with the plasmid encoding green flourescent protein (GFP). Since LTX was more effective transfection agent compared to PEI in all used cell lines, it was used in subsequent experiments. (PDF 417 kb)

Additional file 2: ** Movie.** ORF3 expression causes an increase in abnormal mitosis formation in B16F10 cells. The movie shows that B16F10 cells expressing ORF3 can display chromosome misalignment, disrupted mitotic spindles and abnormal mitosis. (MOV 139 kb)

Additional file 3: **Figure S2.** PCV2 ORF3 Induces Apoptosis in B16F10 Cells through a Caspase-8 and Caspase-3 Independent Pathway. Analysis of caspase-8 and -3 activities of pcDNA3-ORF3 or empty pcDNA3.1 plasmid transfected B16F10 cells at 24 and 48 h post-transfection. pcDNA3-ORF3 24 h (1st bar); pcDNA3-ctr 24 h (2nd bar); pcDNA3-ORF3 48 h (3rd bar); pcDNA3-ctr 48 h (4th bar). Error bars are representative of the standard deviation of triplicates. B: Analysis of caspase-8 and -3 activities of pcDNA3-ORF3 or empty pcDNA3.1 plasmid transfected c57/bl6 mice primary splenocytes at 24 h post-transfection. pcDNA3-ORF3 24 h (1st bar); pcDNA3-ctr 24 h (2nd bar); Non-treated mouse primary splenocytes were used as control (3rd bar); pcDNA3-ORF3 24 h blue bars; pcDNA3-ctr 24 h red bars; Non-treated mouse primary splenocytes - green bars; Error bars are representative of the standard deviation of triplicates. (PDF 496 kb)

Additional file 4: **Figure S3.** PCV2 ORF3 intracellular expression pattern in porcine PBMC. The intracellular localization of PCV2 ORF3 (red) and RGS16 (green, here a counterstaining) was examined in LPS-activated poPBMCs co-transfected with pcDNA3.1-His-ORF3-mCherry and pCEP-GFP-RGS16, then stained with Texas red and FITC 48 h post-transfection. The cells nuclei were stained with the Hoechst 33258 (blue). The cytoplasmic dot-like staining pattern of PCV2 ORF3 is indicated by arrows in all panels. (PDF 1021 kb)
