# Supplementary material for: Porcine circovirus type 2 ORF3 protein induces apoptosis in melanoma cells
Source: BMC Cancer. 2018 Dec 10;18:1237. doi: 10.1186/s12885-018-5090-2 (PMC6288936; doi:10.1186/s12885-018-5090-2)
Supplement: Supplementary file 1 — Figure S1. Transfection efficiency. To find out the most effective way for overexpression of recombinant proteins DNA was introduced into all cell lines by transfection with Lipofectamine (LTX) or polyethyleneimine (PEI). Consequently, the DNA: LTX and DNA: PEI complex penetrate cell membranes and recombinant proteins are produced. After 24–48 h of transfection, cells were harvested, and transfection efficiency of every experiment was determined using parallel wells that were transfected only with the plasmid encoding green flourescent protein (GFP). Since LTX was more effective transfection agent compared to PEI in all used cell lines, it was used in subsequent experiments. (PDF 417 kb) [file 12885_2018_5090_MOESM1_ESM.pdf]

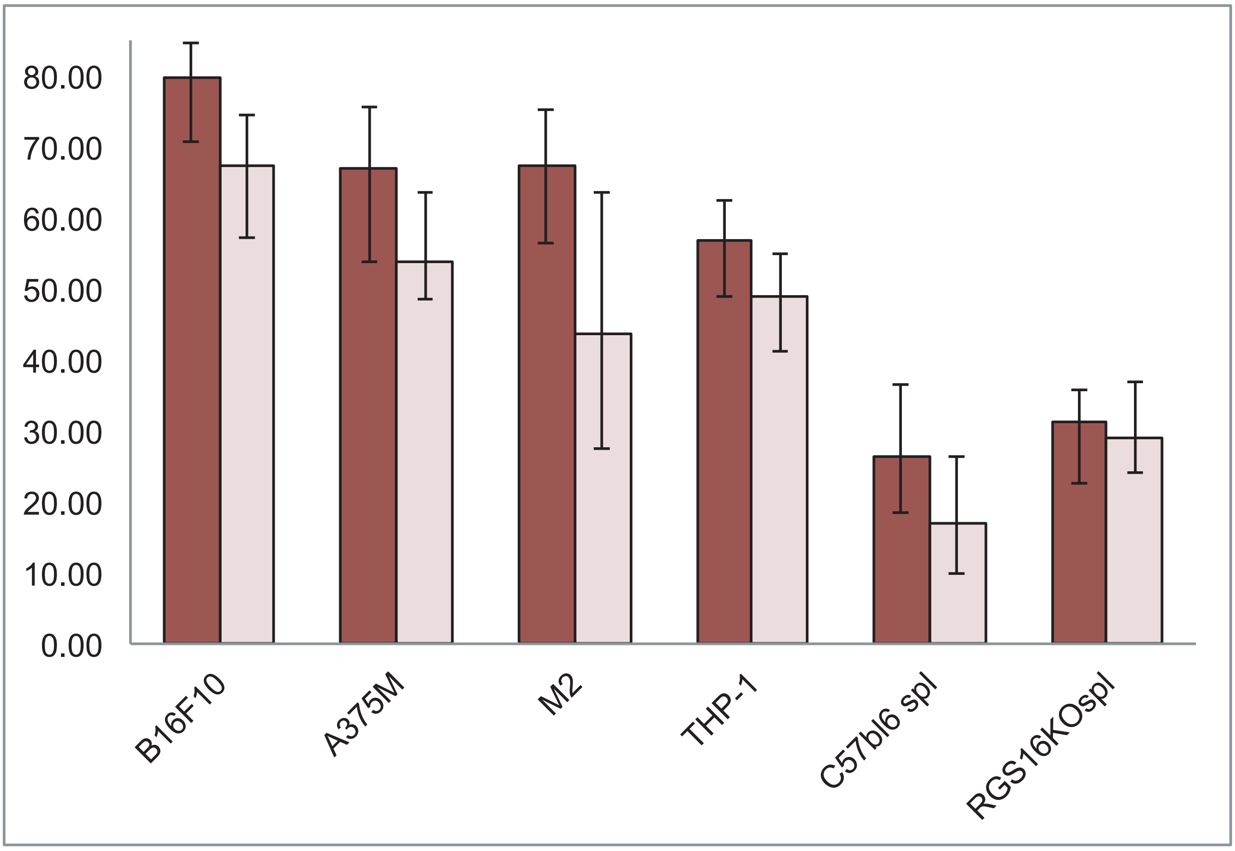

Supplementary Figure 1

Forty eight hours post-transfection with a GFP expressing plasmid, cells were detached with 0.05% trypsin and the fluorescence analysed using a BD FACSCalibur flow cytometer. Thirty thousand events were acquired. Transfection efficacy was estimated by the quantification of the proportion of GFP-positive cells in the sample, as shown for each cell line transfected using lipofectamin (brown bars) or PEI (pink bars).
