# Supplementary material for: Porcine circovirus type 2 ORF3 protein induces apoptosis in melanoma cells
Source: BMC Cancer. 2018 Dec 10;18:1237. doi: 10.1186/s12885-018-5090-2 (PMC6288936; doi:10.1186/s12885-018-5090-2)
Supplement: Supplementary file 3 — Figure S2. PCV2 ORF3 Induces Apoptosis in B16F10 Cells through a Caspase-8 and Caspase-3 Independent Pathway. Analysis of caspase-8 and -3 activities of pcDNA3-ORF3 or empty pcDNA3.1 plasmid transfected B16F10 cells at 24 and 48 h post-transfection. pcDNA3-ORF3 24 h (1st bar); pcDNA3-ctr 24 h (2nd bar); pcDNA3-ORF3 48 h (3rd bar); pcDNA3-ctr 48 h (4th bar). Error bars are representative of the standard deviation of triplicates. B: Analysis of caspase-8 and -3 activities of pcDNA3-ORF3 or empty pcDNA3.1 plasmid transfected c57/bl6 mice primary splenocytes at 24 h post-transfection. pcDNA3-ORF3 24 h (1st bar); pcDNA3-ctr 24 h (2nd bar); Non-treated mouse primary splenocytes were used as control (3rd bar); pcDNA3-ORF3 24 h blue bars; pcDNA3-ctr 24 h red bars; Non-treated mouse primary splenocytes - green bars; Error bars are representative of the standard deviation of triplicates. (PDF 496 kb) [file 12885_2018_5090_MOESM3_ESM.pdf]

A

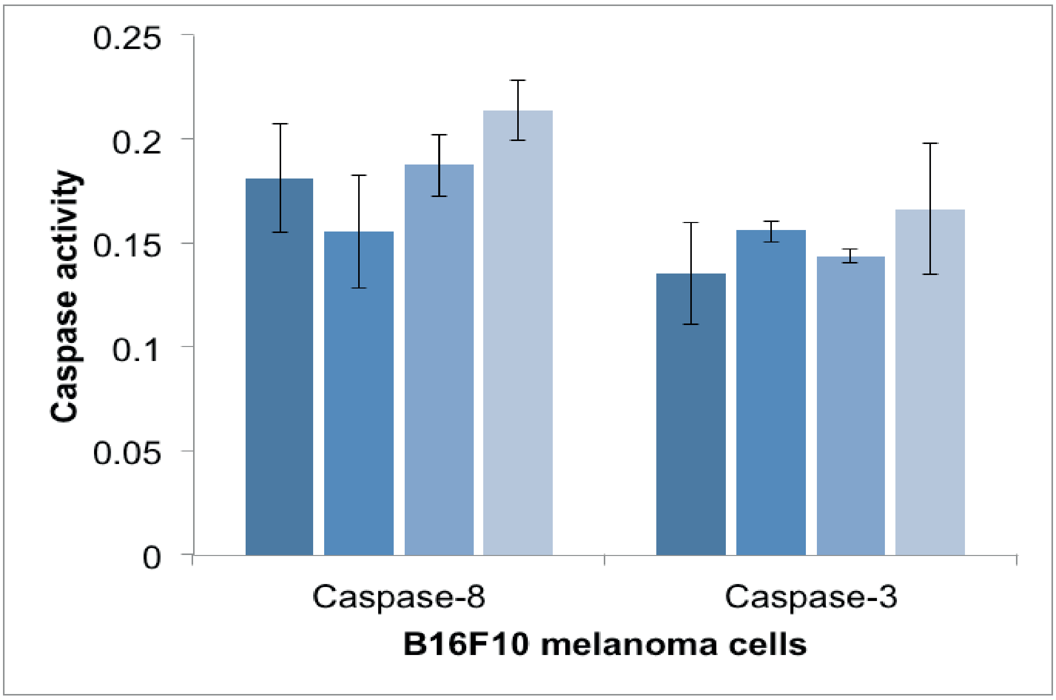

B

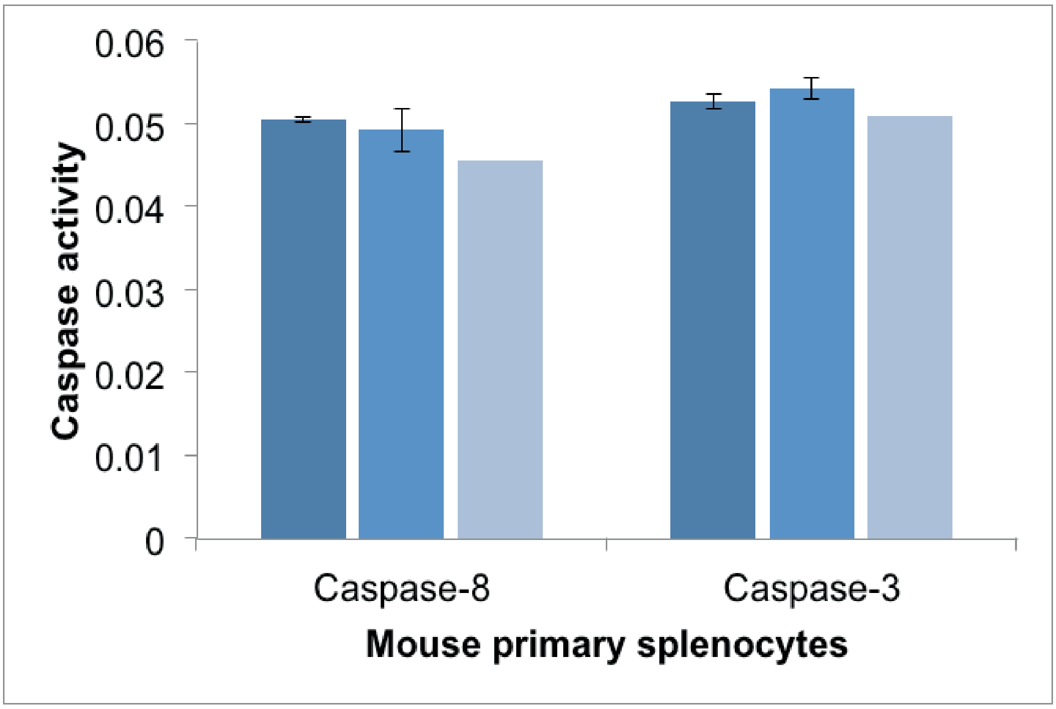

Figure S2. PCV2 ORF3 Induces Apoptosis in B16F10 cells through a Caspase-8 and Caspase-3 Independent Pathway

Analysis of caspase-8 and -3 activities of pcDNA3-ORF3 or empty pcDNA3.1 plasmid transfected B16F10 cells at 24 and 48 hours post-transfection. pcDNA3-ORF3 24h (1st bar); pcDNA3-ctr 24h (2nd bar); pcDNA3-ORF3 48h (3rd bar); pcDNA3-ctr 48h (4th bar). Error bars are representative of standard deviation of triplicates. B: Analysis of caspase-8 and -3 activities of pcDNA3-ORF3 or empty pcDNA3.1 plasmid transfected c57/bl6 mice primary splenocytes at 24 hours post-transfection. pcDNA3-ORF3 24h (1st bar); pcDNA3-ctr 24h (2nd bar); Non-treated mouse primary splenocytes were used as control (3rd bar); Error bars are representative of standard deviation of triplicates.
