# Supplementary material for: Porcine circovirus type 2 ORF3 protein induces apoptosis in melanoma cells
Source: BMC Cancer. 2018 Dec 10;18:1237. doi: 10.1186/s12885-018-5090-2 (PMC6288936; doi:10.1186/s12885-018-5090-2)
Supplement: Supplementary file 4 — Figure S3. PCV2 ORF3 intracellular expression pattern in porcine PBMC. The intracellular localization of PCV2 ORF3 (red) and RGS16 (green, here a counterstaining) was examined in LPS-activated poPBMCs co-transfected with pcDNA3.1-His-ORF3-mCherry and pCEP-GFP-RGS16, then stained with Texas red and FITC 48 h post-transfection. The cells nuclei were stained with the Hoechst 33258 (blue). The cytoplasmic dot-like staining pattern of PCV2 ORF3 is indicated by arrows in all panels. (PDF 1021 kb) [file 12885_2018_5090_MOESM4_ESM.pdf]

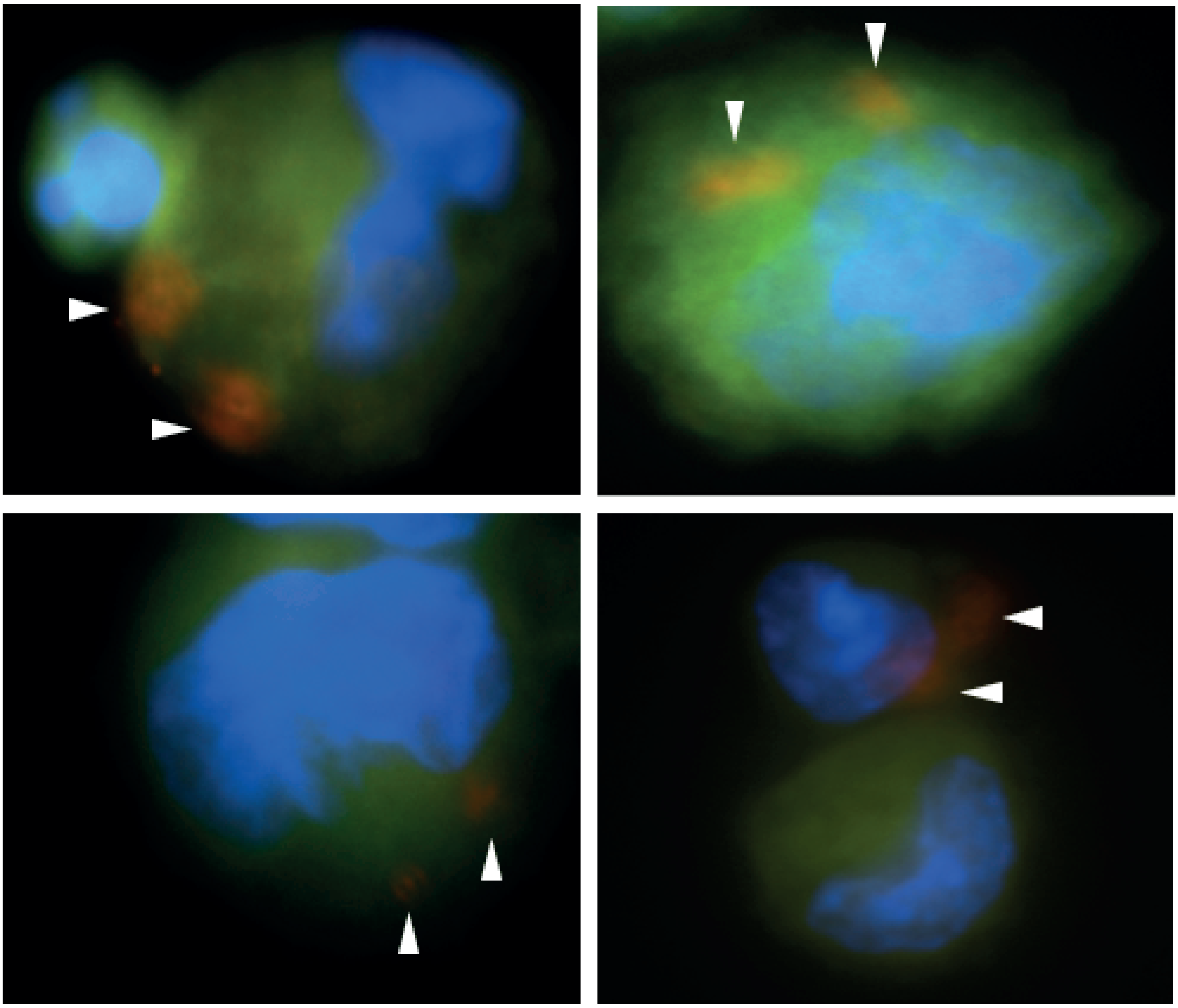

Supplementary Figure 3

Two dot PCV2 ORF3 intracellular expression pattern in porcine 0PBMC. The intracellular localization of PCV2 ORF3 (red) and RGS16 (green, here a counter staining) was examined in LPS-activated poPBMCs co-transfected with pcDNA3.1-His-ORF3-mCherry and pCEP-GFP-RGS16, then stained with Texas red and FITC 48 hours post-transfection.
The cells nuclei were stained with the Hoechst 33258 (blue). Cytoplasmic dot-like staining pattern of PCV2 ORF3 is indicated by arrows in all panels.
